# *catena*-Poly[[[bis­(1*H*-imidazole-κ*N* ^3^)zinc(II)]-μ_2_-imidazol-1-ido-κ^2^ *N*:*N*′] nitrate]

**DOI:** 10.1107/S1600536814015232

**Published:** 2014-07-11

**Authors:** Elumalai Govindhan, A. S. Ganeshraja, B. Bhavana, Krishnamoorthy Anbalagan, Arunachalam SubbiahPandi

**Affiliations:** a Department of Physics, Presidency College, Chennai 600 005, India; b Department of Chemistry, Pondicherry University, Pondicherry 605 014, India; c Department of Physics, Presidency College (Autonomous), Chennai 600 005, India

**Keywords:** crystal structure

## Abstract

The title compound, {[Zn(C_3_H_3_N_2_)(C_3_H_4_N_2_)_2_]NO_3_}_*n*_, is a one-dimensional coordination polymer along [01-1] with the Zn^II^ atom coordinating to four imidazole/imidazolide rings. The Zn^II^ atom has a regular tetra­hedral geometry with the planes of the two monodentate imidazole rings inclined to one another by 87.94 (17)°, while the planes of the bridging imidazolide rings are inclined to one another by 39.06 (17)°. In the crystal, the chains are linked *via* bifurcated N—H⋯(O,O) hydrogen bonds, forming sheets parallel to (001). These two-dimensional networks are linked *via* C—H⋯O hydrogen bonds and a C—H⋯π inter­action, forming a three-dimensional structure.

## Related literature   

For imidazole systems in biological systems, see: Brooks & Davidson, (1960 ▶). For the crystal structure of a similar compound, see: Fu *et al.* (2007 ▶). For the synthesis of the title compound, see: Anbalagan & Lydia (2011 ▶). For standard bond lengths, see: Allen *et al.* (1987 ▶). 
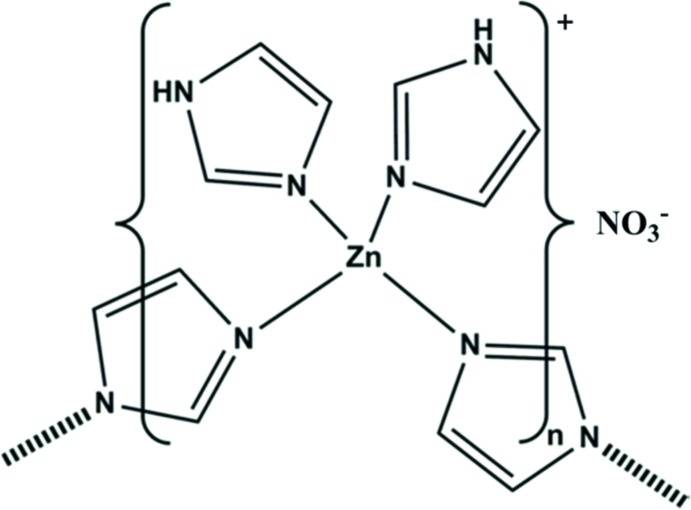


## Experimental   

### 

#### Crystal data  

[Zn(C_3_H_3_N_2_)(C_3_H_4_N_2_)_2_]NO_3_
*M* *_r_* = 330.62
Monoclinic, 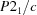

*a* = 12.1812 (10) Å
*b* = 10.0713 (7) Å
*c* = 11.3628 (10) Å
β = 91.011 (8)°
*V* = 1393.78 (19) Å^3^
*Z* = 4
Mo *K*α radiation
μ = 1.78 mm^−1^
*T* = 293 K
0.45 × 0.35 × 0.35 mm

#### Data collection  

Oxford Diffraction Xcalibur Eos diffractometer
Absorption correction: multi-scan (*CrysAlis PRO*; Oxford Diffraction, 2009 ▶) *T*_min_ = 0.478, *T*_max_ = 0.536
7564 measured reflections
3132 independent reflections
2419 reflections with *I* > 2σ(*I*)
*R*_int_ = 0.025

#### Refinement  

*R*[*F* ^2^ > 2σ(*F* ^2^)] = 0.033
*wR*(*F* ^2^) = 0.083
*S* = 1.03
3132 reflections
188 parameters
2 restraints
H atoms treated by a mixture of independent and constrained refinement
Δρ_max_ = 0.26 e Å^−3^
Δρ_min_ = −0.30 e Å^−3^

### 

Data collection: *CrysAlis CCD* (Oxford Diffraction, 2009 ▶); cell refinement: *CrysAlis CCD*; data reduction: *CrysAlis RED* (Oxford Diffraction, 2009 ▶); program(s) used to solve structure: *SHELXS97* (Sheldrick, 2008 ▶); program(s) used to refine structure: *SHELXL2013* (Sheldrick, 2008 ▶); molecular graphics: *PLATON* (Spek, 2009 ▶); software used to prepare material for publication: *SHELXL2013* and *PLATON* (Spek, 2009 ▶).

## Supplementary Material

Crystal structure: contains datablock(s) global, I. DOI: 10.1107/S1600536814015232/su2723sup1.cif

Structure factors: contains datablock(s) I. DOI: 10.1107/S1600536814015232/su2723Isup2.hkl

CCDC reference: 1011008

Additional supporting information:  crystallographic information; 3D view; checkCIF report

## Figures and Tables

**Table 1:** Selected bond lengths (Å)

Zn1—N6	1.9871 (18)
Zn1—N1	1.990 (2)
Zn1—N3	1.994 (2)
Zn1—N5	1.9954 (19)

**Table 2:** Hydrogen-bond geometry (Å, °) *Cg*1 is the centroid of the N1/N2/C1–C3 imidazole ring.

*D*—H⋯*A*	*D*—H	H⋯*A*	*D*⋯*A*	*D*—H⋯*A*
N2—H2*N*⋯O1^i^	0.92 (3)	1.92 (3)	2.809 (3)	162 (3)
N4—H4*N*⋯O1^ii^	0.87 (2)	1.99 (3)	2.826 (3)	161 (3)
N4—H4*N*⋯O3^ii^	0.87 (2)	2.52 (3)	3.074 (3)	122 (2)
C2—H2⋯O3^iii^	0.93	2.37	3.289 (4)	172
C4—H4⋯O3	0.93	2.55	3.283 (4)	135
C7—H7⋯*Cg*1^iv^	0.93	2.88	3.587 (4)	133

Symmetry codes: (i) 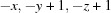; (ii) 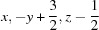; (iii) 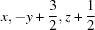; (iv) 
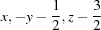
.

**Fig. 1.**
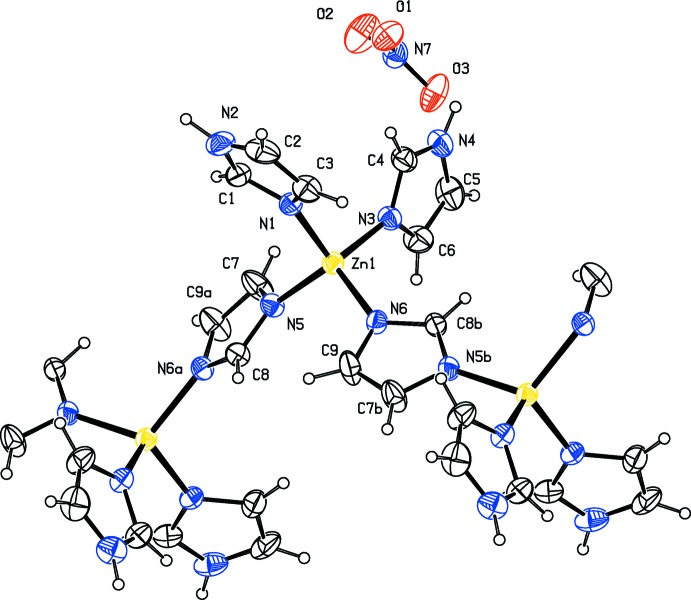
The molecular structure of the title compound, with atomic labelling [Symmetry codes: (a) -x+1, y+1/2, -z+1/2; (b) -x+1, y-1/2, -z+1/2]. Displacement ellipsoids are drawn at the 50% probability level.

**Fig. 2.**
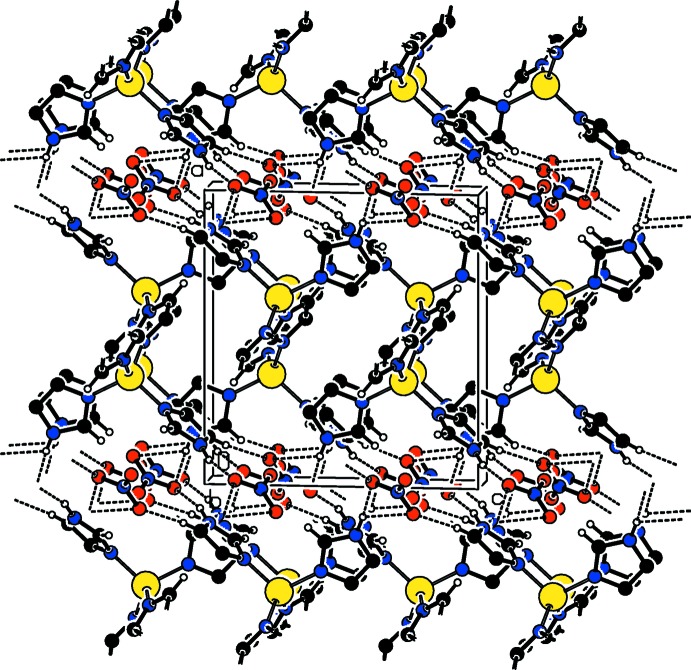
A view along the b-axis of the crystal packing of the title compound. Hydrogen bonds are shown as dashed bonds (see Table 1 for details; H atoms not involved in these interactions have been omitted for clarity).

## supplementary crystallographic information

### S1. Comment

Imidazoles are of considerable interest as ligands in many biological systems as they provide potential binding sites for metal ions (Brooks & Davidson, 1960). Against this background and to ascertain the molecular structure and conformation of the title compound, the crystal structure determination has been carried out.

The molecular structure of the title compound is shown in Fig. 1. Atom Zn1 has a regular tetrahedral geometry and the bond lengths (Allen *et al.*, 1987) and angles are normal. It is a one-dimensional zigzag polymer with atom Zn1 coordinating to four imidazole units. This structure is similar to that observed for the compound catena-(bis(µ2-Imidazole)-tetrakis(1H-imidazole)-di-Zn^II^ 4,4'-bis(2-sulfonatostyryl)biphenyl) [Fu *et al.*, 2007]. Two of the imidazole units are related by a two-fold screw axis and bridge the zinc atoms. The Zn-N bond distances vary from 1.9871 (18) to 1.9954 (19) Å, while the N-Zn-N bond angles vary from 105.86 (2) to 112.65 (8) °. The two monodentate coordinated imidazole rings [N1/N2/C1-C3 and N3/N4/C4-C6] are inclined to one another by 87.94 (17) °, while the bridging imidazole rings [N5/N6^i^/C9/C7^i^/C8^i^ and N5^ii^/N6/C7^ii^/C8^ii^/C9] are inclined to one another by 39.06 (17) ° [symmetry codes: (i) -*x*+1, *y*+1/2, -*z*+1/2; (ii) -*x*+1, *y*-1/2, -*z*+1/2].

In the crystal, the chains are linked via bifurcated N—H···O/O hydrogen bonds forming sheets parallel to (001); (Table 1 and Fig. 2). These two-dimensional networks are linked via C-H···O hydrogen bonds and a C-H···π interaction forming a three-dimensional structure.

### S2. Experimental

The title compound was synthesized following a published procedure (Anbalagan & Lydia, 2011). To an ethanol solution (30 ml) of imidazole (1.0 g, 4.2 mmol) was added an ethanol solution of Zn(NO_3_)_2_.6H_2_O (0.32 g, 1.1 mmol) and the mixture was stirred for 30 min at room temperature. The solvent was removed under vacuum. The white powder obtained was washed several times with water and ether. The final product was dissolved in 5–10 ml of ethanol and allowed to crystallize in a desiccator containing P_2_O_5_ for 4 days. Colourless crystals were obtained [yield > 90%], which were filtered, washed with cold ethanol and dried under vacuum.

### S3. Refinement

NH H atoms were located in a difference Fourier map and refined with distance restraints: N-H = 0.88 (2) Å with U_iso_(H) = 1.2U_eq_(N). The C-bound H atoms were positioned geometrically and allowed to ride on their parent atoms: C–H = 0.93 Å with *U*_iso_(H) = 1.2*U*_eq_(C).

### Crystal data

**Table:**

[Zn(C_3_H_3_N_2_)(C_3_H_4_N_2_)_2_]NO_3_	*F*(000) = 672
*M**_r_* = 330.62	*D*_x_ = 1.576 Mg m^−^^3^
Monoclinic, *P*2_1_/*c*	Mo *K*α radiation, λ = 0.71073 Å
*a* = 12.1812 (10) Å	Cell parameters from 1994 reflections
*b* = 10.0713 (7) Å	θ = 3.9–25.0°
*c* = 11.3628 (10) Å	µ = 1.78 mm^−^^1^
β = 91.011 (8)°	*T* = 293 K
*V* = 1393.78 (19) Å^3^	Block, pink
*Z* = 4	0.45 × 0.35 × 0.35 mm

### Data collection

**Table:**

Oxford Diffraction Xcalibur Eos diffractometer	3132 independent reflections
Radiation source: fine-focus sealed tube	2419 reflections with *I* > 2σ(*I*)
Graphite monochromator	*R*_int_ = 0.025
ω scans	θ_max_ = 29.1°, θ_min_ = 3.9°
Absorption correction: multi-scan (*CrysAlis PRO*; Oxford Diffraction, 2009)	*h* = −16→16
*T*_min_ = 0.478, *T*_max_ = 0.536	*k* = −13→13
7564 measured reflections	*l* = −13→15

### Refinement

**Table:**

Refinement on *F*^2^	Secondary atom site location: difference Fourier map
Least-squares matrix: full	Hydrogen site location: difference Fourier map
*R*[*F*^2^ > 2σ(*F*^2^)] = 0.033	H atoms treated by a mixture of independent and constrained refinement
*wR*(*F*^2^) = 0.083	*w* = 1/[σ^2^(*F*_o_^2^) + (0.0384*P*)^2^ + 0.2989*P*] where *P* = (*F*_o_^2^ + 2*F*_c_^2^)/3
*S* = 1.03	(Δ/σ)_max_ = 0.001
3132 reflections	Δρ_max_ = 0.26 e Å^−^^3^
188 parameters	Δρ_min_ = −0.30 e Å^−^^3^
2 restraints	Extinction correction: *SHELXL2013* (Sheldrick, 2008), Fc^*^=kFc[1+0.001xFc^2^λ^3^/sin(2θ)]^-1/4^
Primary atom site location: structure-invariant direct methods	Extinction coefficient: 0.0081 (9)

### Special details

**Table:**

Geometry. All esds (except the esd in the dihedral angle between two l.s. planes) are estimated using the full covariance matrix. The cell esds are taken into account individually in the estimation of esds in distances, angles and torsion angles; correlations between esds in cell parameters are only used when they are defined by crystal symmetry. An approximate (isotropic) treatment of cell esds is used for estimating esds involving l.s. planes.

### Fractional atomic coordinates and isotropic or equivalent isotropic displacement parameters (Å^2^)

**Table:**

	*x*	*y*	*z*	*U*_iso_*/*U*_eq_	
Zn1	0.36252 (2)	0.46682 (2)	0.22841 (2)	0.03923 (12)	
N1	0.25332 (15)	0.47069 (19)	0.35663 (18)	0.0432 (5)	
N2	0.13625 (19)	0.4040 (3)	0.4868 (2)	0.0660 (7)	
H2N	0.087 (2)	0.347 (3)	0.521 (3)	0.079*	
N3	0.28898 (17)	0.5089 (2)	0.07477 (18)	0.0453 (5)	
N4	0.1623 (2)	0.5621 (2)	−0.0543 (2)	0.0629 (6)	
H4N	0.0994 (18)	0.591 (3)	−0.081 (3)	0.075*	
N5	0.42417 (16)	0.28445 (18)	0.21108 (18)	0.0445 (5)	
N6	0.47785 (15)	0.59804 (18)	0.27271 (17)	0.0417 (4)	
C1	0.1922 (2)	0.3708 (3)	0.3939 (2)	0.0542 (7)	
H1	0.1894	0.2875	0.3585	0.065*	
C2	0.1596 (2)	0.5311 (4)	0.5132 (3)	0.0699 (9)	
H2	0.1314	0.5802	0.5750	0.084*	
C3	0.2327 (2)	0.5742 (3)	0.4320 (3)	0.0591 (7)	
H3	0.2632	0.6587	0.4282	0.071*	
C4	0.1872 (2)	0.5504 (3)	0.0592 (3)	0.0547 (7)	
H4	0.1392	0.5689	0.1198	0.066*	
C5	0.2504 (3)	0.5270 (3)	−0.1158 (3)	0.0730 (9)	
H5	0.2564	0.5264	−0.1973	0.088*	
C6	0.3286 (3)	0.4929 (3)	−0.0364 (3)	0.0642 (8)	
H6	0.3986	0.4632	−0.0542	0.077*	
C7	0.3839 (3)	0.1932 (3)	0.1340 (3)	0.0754 (10)	
H7	0.3248	0.2063	0.0824	0.090*	
C8	0.50637 (19)	0.2215 (2)	0.2642 (2)	0.0445 (6)	
H8	0.5497	0.2606	0.3227	0.053*	
C9	0.5567 (3)	0.5799 (3)	0.3560 (3)	0.0733 (10)	
H9	0.5684	0.5026	0.3993	0.088*	
O1	−0.02383 (16)	0.7857 (2)	0.38003 (18)	0.0670 (5)	
O2	−0.0378 (2)	0.6253 (3)	0.2556 (2)	0.0974 (8)	
O3	0.0817 (2)	0.7775 (2)	0.23084 (19)	0.0834 (7)	
N7	0.00643 (19)	0.7290 (2)	0.2867 (2)	0.0568 (6)	

### Atomic displacement parameters (Å^2^)

**Table:**

	*U*^11^	*U*^22^	*U*^33^	*U*^12^	*U*^13^	*U*^23^
Zn1	0.04116 (18)	0.03470 (16)	0.04196 (18)	0.00129 (11)	0.00404 (12)	−0.00016 (11)
N1	0.0413 (11)	0.0480 (11)	0.0405 (11)	0.0025 (9)	0.0064 (9)	0.0014 (9)
N2	0.0451 (13)	0.097 (2)	0.0562 (16)	−0.0008 (13)	0.0111 (12)	0.0142 (15)
N3	0.0462 (11)	0.0477 (11)	0.0420 (12)	0.0069 (9)	0.0037 (9)	0.0008 (9)
N4	0.0626 (16)	0.0636 (15)	0.0620 (16)	0.0042 (12)	−0.0114 (14)	0.0139 (12)
N5	0.0454 (11)	0.0360 (10)	0.0520 (12)	0.0020 (8)	−0.0039 (10)	−0.0049 (9)
N6	0.0455 (11)	0.0358 (9)	0.0436 (11)	−0.0035 (8)	0.0004 (9)	0.0027 (9)
C1	0.0436 (14)	0.0641 (16)	0.0549 (16)	−0.0020 (12)	0.0030 (13)	0.0091 (13)
C2	0.0556 (17)	0.108 (3)	0.0462 (17)	0.0285 (17)	0.0086 (14)	−0.0098 (17)
C3	0.0615 (17)	0.0609 (16)	0.0552 (17)	0.0117 (13)	0.0055 (14)	−0.0091 (14)
C4	0.0530 (16)	0.0567 (16)	0.0544 (17)	0.0098 (12)	0.0033 (13)	0.0038 (13)
C5	0.096 (3)	0.082 (2)	0.0402 (16)	0.0060 (19)	−0.0029 (17)	0.0034 (15)
C6	0.0610 (17)	0.081 (2)	0.0515 (17)	0.0143 (14)	0.0134 (15)	−0.0019 (14)
C7	0.085 (2)	0.0483 (15)	0.091 (2)	0.0114 (14)	−0.0504 (19)	−0.0149 (16)
C8	0.0456 (13)	0.0385 (12)	0.0492 (14)	0.0000 (10)	−0.0062 (11)	−0.0063 (11)
C9	0.097 (2)	0.0430 (14)	0.078 (2)	−0.0117 (15)	−0.0373 (19)	0.0206 (15)
O1	0.0679 (12)	0.0734 (13)	0.0602 (12)	−0.0107 (10)	0.0150 (10)	−0.0139 (11)
O2	0.117 (2)	0.0852 (17)	0.0903 (18)	−0.0450 (15)	0.0065 (15)	−0.0264 (14)
O3	0.1114 (17)	0.0825 (15)	0.0574 (13)	−0.0286 (14)	0.0313 (13)	0.0009 (12)
N7	0.0637 (14)	0.0596 (14)	0.0469 (12)	−0.0080 (12)	−0.0052 (12)	0.0048 (11)

### Geometric parameters (Å, º)

**Table:**

Zn1—N6	1.9871 (18)	C1—H1	0.9300
Zn1—N1	1.990 (2)	C2—C3	1.364 (4)
Zn1—N3	1.994 (2)	C2—H2	0.9300
Zn1—N5	1.9954 (19)	C3—H3	0.9300
N1—C1	1.325 (3)	C4—H4	0.9300
N1—C3	1.375 (3)	C5—C6	1.345 (4)
N2—C1	1.310 (4)	C5—H5	0.9300
N2—C2	1.344 (4)	C6—H6	0.9300
N2—H2N	0.915 (18)	C7—C9^ii^	1.355 (4)
N3—C4	1.317 (3)	C7—H7	0.9300
N3—C6	1.369 (3)	C8—N6^ii^	1.327 (3)
N4—C4	1.325 (4)	C8—H8	0.9300
N4—C5	1.339 (4)	C9—C7^i^	1.355 (4)
N4—H4N	0.872 (17)	C9—H9	0.9300
N5—C8	1.322 (3)	O1—N7	1.265 (3)
N5—C7	1.355 (3)	O2—N7	1.224 (3)
N6—C8^i^	1.327 (3)	O3—N7	1.226 (3)
N6—C9	1.349 (3)		
			
N6—Zn1—N1	106.27 (8)	N2—C2—C3	106.9 (3)
N6—Zn1—N3	112.65 (8)	N2—C2—H2	126.6
N1—Zn1—N3	109.94 (8)	C3—C2—H2	126.6
N6—Zn1—N5	111.81 (8)	C2—C3—N1	108.0 (3)
N1—Zn1—N5	110.35 (8)	C2—C3—H3	126.0
N3—Zn1—N5	105.86 (8)	N1—C3—H3	126.0
C1—N1—C3	105.5 (2)	N3—C4—N4	111.0 (3)
C1—N1—Zn1	127.24 (18)	N3—C4—H4	124.5
C3—N1—Zn1	127.06 (19)	N4—C4—H4	124.5
C1—N2—C2	108.1 (3)	N4—C5—C6	106.3 (3)
C1—N2—H2N	122 (2)	N4—C5—H5	126.8
C2—N2—H2N	130 (2)	C6—C5—H5	126.8
C4—N3—C6	105.0 (2)	C5—C6—N3	109.4 (3)
C4—N3—Zn1	126.31 (19)	C5—C6—H6	125.3
C6—N3—Zn1	128.51 (18)	N3—C6—H6	125.3
C4—N4—C5	108.2 (3)	N5—C7—C9^ii^	109.3 (2)
C4—N4—H4N	124 (2)	N5—C7—H7	125.3
C5—N4—H4N	128 (2)	C9^ii^—C7—H7	125.3
C8—N5—C7	103.4 (2)	N5—C8—N6^ii^	114.7 (2)
C8—N5—Zn1	132.85 (15)	N5—C8—H8	122.6
C7—N5—Zn1	123.74 (16)	N6^ii^—C8—H8	122.6
C8^i^—N6—C9	104.1 (2)	N6—C9—C7^i^	108.5 (2)
C8^i^—N6—Zn1	130.45 (16)	N6—C9—H9	125.8
C9—N6—Zn1	125.38 (17)	C7^i^—C9—H9	125.8
N2—C1—N1	111.5 (3)	O2—N7—O3	121.4 (3)
N2—C1—H1	124.2	O2—N7—O1	119.6 (2)
N1—C1—H1	124.2	O3—N7—O1	119.1 (2)
			
C2—N2—C1—N1	0.4 (3)	C4—N4—C5—C6	0.5 (4)
C3—N1—C1—N2	−0.6 (3)	N4—C5—C6—N3	−0.9 (4)
Zn1—N1—C1—N2	174.19 (16)	C4—N3—C6—C5	0.9 (3)
C1—N2—C2—C3	−0.1 (3)	Zn1—N3—C6—C5	176.7 (2)
N2—C2—C3—N1	−0.3 (3)	C8—N5—C7—C9^ii^	0.0 (4)
C1—N1—C3—C2	0.5 (3)	Zn1—N5—C7—C9^ii^	−179.8 (2)
Zn1—N1—C3—C2	−174.26 (18)	C7—N5—C8—N6^ii^	0.2 (3)
C6—N3—C4—N4	−0.6 (3)	Zn1—N5—C8—N6^ii^	179.99 (17)
Zn1—N3—C4—N4	−176.58 (18)	C8^i^—N6—C9—C7^i^	0.3 (4)
C5—N4—C4—N3	0.1 (3)	Zn1—N6—C9—C7^i^	−176.6 (2)

Symmetry codes: (i) −*x*+1, *y*+1/2, −*z*+1/2; (ii) −*x*+1, *y*−1/2, −*z*+1/2.

### Hydrogen-bond geometry (Å, º)

Cg1 is the centroid of the N1/N2/C1–C3 imidazole ring.

**Table:**

*D*—H···*A*	*D*—H	H···*A*	*D*···*A*	*D*—H···*A*
N2—H2*N*···O1^iii^	0.92 (3)	1.92 (3)	2.809 (3)	162 (3)
N4—H4*N*···O1^iv^	0.87 (2)	1.99 (3)	2.826 (3)	161 (3)
N4—H4*N*···O3^iv^	0.87 (2)	2.52 (3)	3.074 (3)	122 (2)
C2—H2···O3^v^	0.93	2.37	3.289 (4)	172
C4—H4···O3	0.93	2.55	3.283 (4)	135
C7—H7···*Cg*1^vi^	0.93	2.88	3.587 (4)	133

Symmetry codes: (iii) −*x*, −*y*+1, −*z*+1; (iv) *x*, −*y*+3/2, *z*−1/2; (v) *x*, −*y*+3/2, *z*+1/2; (vi) *x*, −*y*−1/2, *z*−3/2.
